# Learning biomolecular absorption spectra in graphene nanopores

**DOI:** 10.1039/d6ra03783f

**Published:** 2026-07-29

**Authors:** Longlong Li, Maria Fyta

**Affiliations:** a Computational Biotechnology, RWTH Aachen University Worrignerweg 3 Aachen 52074 Germany l.li@biotec.rwth-aachen.de; b Center for Computational Life Sciences (CCLS), RWTH Aachen University Pauwelsstrasse 19 Aachen 52072 Germany

## Abstract

Solid-state nanopores can be combined with optical measurements for the detection of biomolecules. With a view to a reliable optical biosensing library and avoiding computationally demanding simulations or expensive experiments, we develop a learning approach based on deep neural networks that are being trained on electronic, conformational, and optical characteristics from density functional theory (DFT) simulations of single amino acids placed in a graphene nanopore. Principal component analysis is used to compress the high-dimensional data into a latent representation, improving training efficiency while preserving the most important physical features. For the five amino acids considered in this work, the learning model predicts the full orientation-dependent absorption spectra with consistently high accuracy in previously unseen molecular orientations within each amino acid dataset, with test *R*^2^ values exceeding 0.9. These results indicate robust interpolation across rich conformational variations of the exact amino acids studied here. The developed workflow enables the construction of reliable optical biosensing libraries with near-DFT accuracy at a fraction of the computational cost. It thus provides a scalable and efficient pathway towards real-time, high-precision optical identification of biomolecules.

## Abstract 2

Neural networks trained on density functional theory data from 3D-rotated amino acids in graphene nanopores accurately predict molecular optical fingerprints for rapid sensing applications.

## Graphical abstract

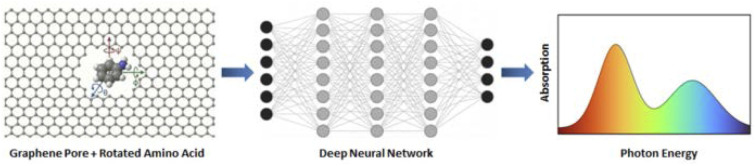

## Introduction

Nanopore biosensing has emerged as one of the most promising technologies for single-molecule detection and analysis.^1^ By monitoring ionic or transverse current signals generated during the translocation of molecules through a nanoscale opening, nanopores enable direct interrogation of the molecular fingerprints of DNA, RNA, and proteins at the single-molecule level.^2,3^ This capability has become the basis of next-generation sequencing technologies and has significantly influenced fields such as genomics, epigenetics, and precision medicine.^4^

Biological nanopores, including α-hemolysin and *Mycobacterium smegmatis* porin A (MspA), have demonstrated remarkable sequencing accuracy and remain central to current nanopore sequencing technologies.^5,6^ Representative commercial implementations include the MinION and GridION platforms developed by Oxford Nanopore Technologies, which use biological nanopores for long-read DNA and RNA sequencing.^7,8^ In parallel, solid-state nanopores have attracted extensive interest due to their mechanical robustness, controllable geometry, and integrated compatibility with electronic devices.^9,10^ In particular, nanopores in two-dimensional (2D) materials such as graphene and molybdenum disulfide (MoS_2_) have received significant attention because of their intrinsic atomic-scale thickness,^11,12^ thereby providing the theoretical possibility of resolving single-base detection of DNA molecules. Recent representative studies further highlight the broadening scope of nanopore biosensing, including single-molecule detection of amyloid-β aggregation in confined glass nanopores,^13^ direct tracking of transient peptide-folding pathways with solid-state nanopores,^14^ single-molecule protein detection from single cells using solid-state nanopores,^15^ and the wider analytical framework of nanopipette electrochemistry for nanoscale sensing.^16^ Despite these advances, reliable nanopore biosensing remains challenging, mainly because detection signals often have interference from unwanted noise,^17,18^ making molecular identification very difficult. Moreover, the interpretation of those signals at the single-molecule level is also very complex due to the similarity in their histogram plots and the massive information they contain.^3^ Motivated by this, advanced computational approaches combined with data-driven methods have emerged as powerful strategies capable of analyzing noisy, complex, and massive signal data that are difficult to resolve using conventional analytical approaches.^19,20^

In recent years, the integration of Machine Learning (ML) with nanoscale sequencing architectures has rapidly evolved across nanogaps, nanopores, and nanochannels.^21^ Early pioneering work demonstrated that support vector machine classifiers could distinguish DNA nucleobases from stochastic recognition tunneling signals obtained using functionalized gold nanogap electrodes, achieving up to 95% accuracy when multiple current spikes were analyzed.^22^ Subsequent investigation further demonstrated that frequency domain characteristics of Brownian fluctuations could enable ML-based nucleobase recognition under realistic solvent conditions.^23^ Beyond nanogap architectures, solid-state nanopores have been extensively integrated with unsupervised and supervised learning models.^24–26^ For instance, *k*-means clustering of ionic blockade features, particularly blockade height rather than dwell time, enabled label-free differentiation of single-nucleotide events in MoS_2_ nanopores.^24^ Deep neural networks, convolutional neural networks, and long short-term memory models subsequently improved readout efficiency, reaching approximately 94% classification accuracy through dimensionality-reduced features.^25^

A hybrid workflow combining supervised and unsupervised methods enabled accurate single-molecule identification of multiple antiviral and anticancer nucleoside drugs using nanopore signal readouts.^26^ In parallel, quantum transport simulations combined with regression and classification models such as random forest classifiers and extreme gradient boosting regressors, enabling high precision prediction of transmission spectra and nucleotide class identification in gold, graphene, phosphorene, C_3_N, and hybrid nanopores.^27–32^ In addition, the introduction of chemically informed descriptors such as SMILES- and RDKit-based fingerprints into the XGBoost regression algorithm reduced mean absolute errors in MoS_2_ nanochannel-based sequencing.^33^ In biological nanopores, neural network architectures have extended molecular alphabets to include chemically modified bases, enabling expanded data storage and improved epigenetic detection.^34^ Together, these studies reveal several important themes: feature engineering is pivotal for discriminating complex signal variations, ML can operate effectively in both experimental and theoretical regimes and across both low and large data conditions, and integration of physics-based modeling with data-driven ML substantially enhances predictive efficiency. Nevertheless, the majority of reported work has focused on ionic current blockade or electronic transmission signatures, whereas optical absorption-based biosensing in 2D nanopores remains comparatively unexplored within combined density functional theory (DFT) and ML frameworks.

Despite significant progress in ML-assisted nanopore sequencing, the predictive modeling of molecular absorption spectra in atomically thin nanopore platforms, particularly graphene, remains comparatively underexplored. Electrical transport measurements provide valuable information, yet they often suffer from electrode–molecule coupling, and this requires complex device fabrication. By contrast, optical absorption spectroscopy eliminates the need for electrodes, avoiding issues related to complex device fabrication. Moreover, it can exhibit distinct spectral features, especially in graphene where plasmonic and interband transitions are strongly modulated by molecular adsorption. However, absorption signatures of molecules confined within graphene nanopores are strongly configuration-dependent, making deterministic detection and analysis challenging. For instance, when confined within a nanopore, a single amino acid is not stationary. It undergoes continuous rotation, leading to a broad range of spatial orientations, and each orientation (configuration) generates a distinct absorption spectrum, forming a configuration-dependent optical fingerprint.

To date, ML has not been well leveraged for decoding and predicting molecular absorption spectra in nanopores in the area of single-molecule identification. In this work, we address this need by developing a physics-informed DFT + ML framework that combines first-principles DFT calculations with data-driven ML algorithms to learn and predict optical absorption fingerprints of single amino acids confined in graphene nanopores. Using DFT-derived electronic structure and optical absorption data as the ground truth, we construct physically meaningful descriptors, including structural and electronic features, to efficiently train deep neural network models for predicting configuration-dependent absorption spectra with high accuracy. By integrating atomically thin graphene nanopores with ML models, our work expands nanopore biosensing beyond ML-assisted electrical protocols such as ionic current blockade and transverse electronic current, introducing optical absorption spectroscopy as an alternative data-driven modality for single-molecule recognition.

## Methods

### Learning scheme and data

The data used for the learning process were generated using our recent DFT simulations.^35^ With these, we have modelled the conformational variability of single amino acids in a graphene nanopore and calculated the absorption spectra for the combined molecule/material system.^35^ Specifically, five selected amino acids, the basic aromatic (His), different sizes of alkyl-chain non-polar (Ala, Gly, Val), and the non-polar aromatic (Phe) were placed in a graphene nanopore of 1.5 nm diameter. The conformational space of each amino acid in the nanopore was sampled. For this, the amino acids were rotated within the pore with respect to the 3D rotation angles *θ*, *ϕ*, and *ψ* around the *x*, *y*, and *z* coordination axes, respectively. For all of these configurations, the optical absorption *α*(*ω*) as a function of the light frequency (*ω*) was calculated from the complex dielectric function using DFT and the linear response theory. The structural and electronic data generated from the DFT calculations^35^ were used to train a neural network model capable of predicting the orientation-dependent absorption spectrum of an amino acid confined within a graphene nanopore. In the present implementation, the model is trained separately for each amino acid rather than jointly across all five amino acids.

**Fig. 1:**
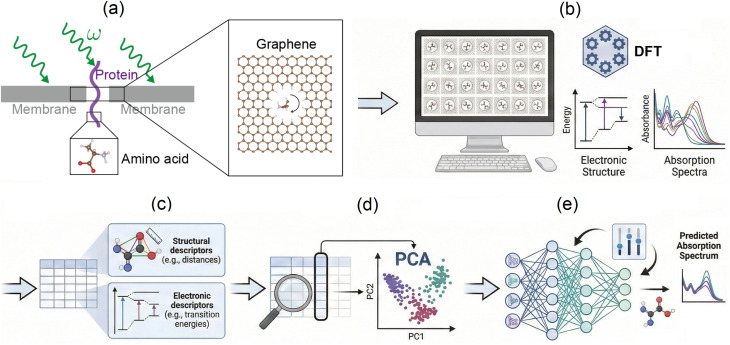
The (DFT + ML) workflow combining DFT and ML to predict molecular absorption spectra in graphene nanopores. (a) A single amino acid rotates within a graphene nanopore, generating diverse molecular orientations. (b) High-throughput DFT simulations compute electronic structures and corresponding absorption spectra for selected molecular configurations. (c) Structural (interatomic distances) and electronic (transition energies) descriptors (features) are extracted from simulation data as input features. (d) Principal component analysis is applied to reduce the dimensionality of absorption spectral data as output targets. (e) A deep neural network is trained to predict absorption spectra for unseen molecular orientations across different amino acids.

The overall workflow consists of four stages, as sketched in Fig. 1. First, a graphene nanopore was constructed for optical biosensing simulation, in which a confined amino acid undergoes continuous rotation inside the pore, generating distinct spatial molecular orientations (configurations). Second, high-throughput DFT simulations were performed previously on a representative subset of molecular configurations to compute electronic structures and corresponding absorption spectra.^35^ Third, physically meaningful descriptors are extracted here from the DFT-simulated results, specifically structural and electronic descriptors, as will be mentioned below, and principal component analysis (PCA) was applied to compress the high-dimensional absorption spectra. Finally, a deep neural network (DNN) model, specifically a multilayer perceptron (MLP), was trained to learn the mapping between structural and electronic features and absorption spectra, enabling rapid prediction of the optical absorption spectra for previously unseen molecular orientations.

### Features and pre-processing

Each sample conformation of a single amino acid in the pore is represented by 303 input features derived from the DFT calculations, as shown in Table 1. These features consist of three orientation angles (*θ*, *ϕ*, *ψ*), 100 structural descriptors (interatomic distances), and 200 electronic descriptors (transition energies). The interatomic-distance features are constructed from the distances between the amino-acid atoms (C, H, O, and N) and the carbon atoms at the pore edge. These distances are sorted in ascending order, and the first 100 smallest values are retained. The transition-energy features are taken from the valence-to-conduction electronic transitions, sorted in ascending energy, and the first 200 lowest-energy transitions are retained. No additional feature-selection algorithm is applied after this physically motivated truncation. All retained features are used directly in the learning model. The structural descriptors are closely relevant to the geometric configuration of an amino acid confined within the nanopore, while the electronic descriptors are closely relevant to the optical response of the system. By combining both descriptors, the feature space captures both geometric confinement effects and orientation-dependent optical response. No additional feature-selection algorithm is applied after this physically motivated descriptor construction; all retained features are used directly in the learning model.

**Table 1:** Summary of input physical features used in the model

Feature	Description
Orientation angles	The three rotation angles (*θ*, *ϕ*, *ψ*) describing the molecular orientation inside the nanopore
Inter-atomic distances	Real-space distances between the C, H, O, and N atoms of amino acid and the C atoms at the pore edge. The distances are sorted in ascending order and the first 100 smallest values are retained as features
Transition energies	Energies corresponding to electronic transitions from lower valence bands to higher conduction bands. The energies are sorted in ascending order and the first 200 lowest values are selected as features

**Fig. 2:**
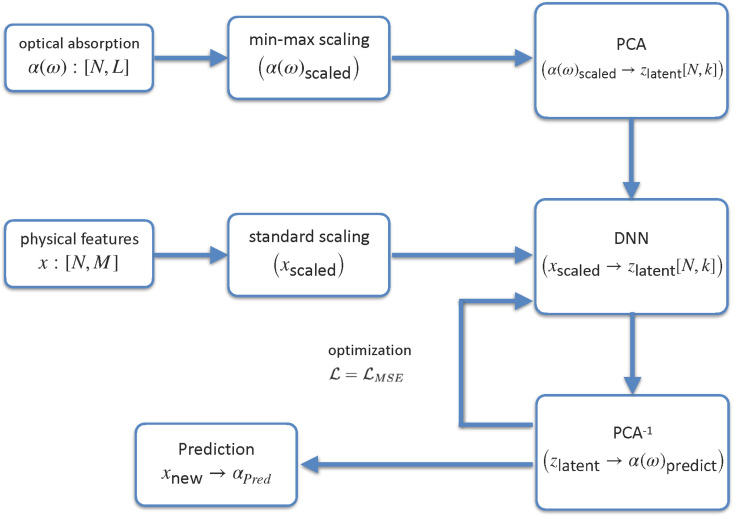
The ML pipeline for predicting molecular absorption spectra. Input physical (structural and electronic) features of dimension (*N*, *M*) are standardized (using the standard scaling) and fed into a deep neural network, where *N* and *M* are the number of samples (molecular configurations) and the number of features per sample, respectively. The target optical spectra (*α*(*ω*)) of dimension (*N*, *L*) are first normalized (using the min–max scaling) and then compressed through a principal component analysis (PCA), where *L* is the number of photon energies. The network (DNN) is trained to learn the mapping from the standardized input features to the normalized and PCA-compressed spectra. During inference, the predicted PCA components are transformed back to the original spectral space *via* inverse PCA (PCA^−1^). The loss function of mean squared error (MSE) 
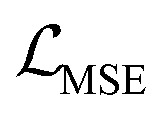
 is used to optimize the DNN through an iterative minimization process before deploying the DNN model to predict the absorption spectra based on new (unseen) input features (*x*_new_).

The ML pipeline for predicting molecular absorption spectra is shown in Fig. 2. The original absorption spectra computed by DFT contain 2000 photon energy points per sample, forming a high-dimensional output space. The data are split into training and test subsets. First, to improve numerical stability during the training, data scaling techniques were applied to both input physical features (constructed by inter-atomic distances and electronic transition energies) and target absorption spectra. Specifically, input features were standardized using the standard scaling, while target spectra were normalized using the min–max scaling. The input standardization, target normalization, and PCA transformation are fitted using the training data only and subsequently applied to the test data. In order to exclude the possibility of data leakage due to the PCA-applied compression of the absorption spectra, we have performed a benchmark by applying the PCA to the whole data set (training and testing) at the same split. For the representative ALA case, the mean test *R*^2^ from the clean and the leaky workflows is 0.9900 and 0.9899, respectively, leading to a difference of only 10^−4^. Such a negligible difference was confirmed across all five amino acids. Accordingly, the performance of our model is not artificially inflated by PCA leakage (refer to Fig. 1 of the SI for the sample-resolved clean-*versus*-leaky comparison). Next, to improve training efficiency and remove learning noise, PCA was applied to compress the normalized target spectra. The first 10 principal components were retained, preserving 99.5% of the total spectral variance. Accordingly, the choice of a 10-dimensional latent representation is guided by the cumulative explained variance rather than by an arbitrary fixed threshold. This dimensionality reduction generates a compact latent representation of the spectral data while maintaining essential physical information. The standard scaling, min–max scaling, and PCA were implemented using the scikit-learn library.^36^

### Neural network architecture

The predictive model is implemented as a fully connected MLP.^37^ The architecture consists of three hidden layers, each containing 128 neurons. A rectified linear unit (ReLU) activation function is applied after each hidden layer to introduce nonlinearity, enabling the network to capture the complex relationships between molecular configurations and their corresponding optical responses. Following each activation, dropout regularization is employed to prevent model overfitting and reduce neuron dependence, enabling the network to learn more robust feature representations.

The model takes a 303-dimensional standardized feature vector as its input, comprising the three orientation angles, the interatomic-distance descriptors, and the transition-energy descriptors, and predicts a reduced 10-dimensional PCA representation of the absorption spectrum as output. The training was conducted over 1000 epochs using the designated training dataset, allowing the network to learn the mapping from physically informed features to the compressed absorption spectra. The procedure utilizes the mean squared error (MSE) as the loss function. The weight and bias parameters are optimized using the Adam optimizer with a learning rate of 0.001. During inference, the predicted principal component coefficients are transformed back into the original spectral domain through an inverse PCA transformation, thereby reconstructing the full absorption spectrum consisting of 2000 data points.

## Results and discussion

The nanopore data used in this work for both input features (inter-atomic distances and electronic transition energies) and output targets (optical absorption spectra) are generated by performing high-throughput DFT simulations on selected molecular configurations of single amino acids within a graphene nanopore.^35^ Specifically, the dataset obtained from high-throughput DFT simulations comprised 1728 samples per amino acid, corresponding to systematic rotational sampling along three angular degrees of freedom (Δ*θ* = Δ*ϕ* = Δ*ψ* = 30°). The data were split into training and test (unseen) sets, with the test size of 10%, leading to 1555 training samples and 173 test samples. Model performance was evaluated using the *R*^2^ score, which quantifies or measures the agreement between ML-predicted and DFT-computed absorption spectra. In the present work, the transition energies are DFT-derived descriptors, and the current ML model should therefore be viewed as a surrogate model for rapidly predicting the absorption spectra once these physically informed descriptors are available. In other words, the present framework accelerates the generation of optical spectra relative to direct high-throughput DFT calculations, but it does not fully eliminate the need for first-principles input features. We have further assessed the robustness of the model performance using a 5-fold cross-validation and an angle-block validation scheme, in which entire groups of one angular coordinate were held out from training.

### Learning assessment

**Fig. 3:**
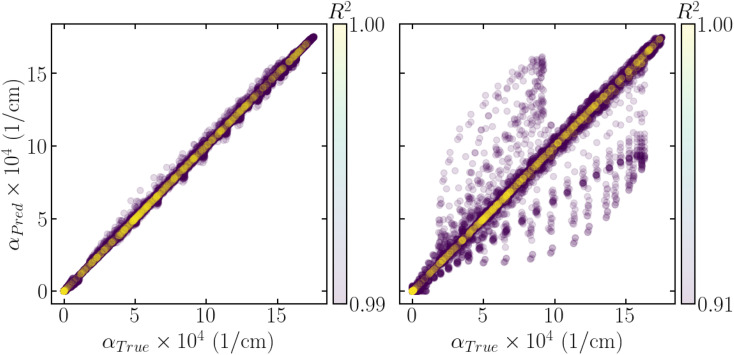
The *R*^2^ score for the (left) training and (right) the testing process in the case of ALA in the nanopore, with 1555 (173) samples in the training (testing) data. The graphs show the correlation of the true and predicted absorption spectra, *α*_True_ and *α*_Pred_, respectively.

The assessment of the learning model is depicted in Fig. 3 for the representative case of an alanine (ALA) amino acid confined within the graphene nanopore. As can be seen in the figure, the predicted absorption spectra, *α*_Pred_, exhibit a strong linear correlation with the corresponding reference spectra, *α*_True_, indicating that the model successfully captures the underlying relationship between molecular configuration and optical response. The linear distribution of data points along the diagonal indicates not only a high degree of predictive accuracy but also that systematic deviations are minimal across the sampled configurations. Although a limited number of points deviate from the diagonal in the testing results, as shown on the right-hand side of the figure, these off-diagonal distributions remain tightly gathered, thereby preserving a high *R*^2^ score of exceeding 0.90. This suggests that the model exhibits a fairly robust performance even for previously unseen molecular configurations, where variations in structural geometry and electronic structure could, in principle, introduce additional complexity. The persistent high *R*^2^ scores indicate that (i) the model learns the mapping from molecular configurations to absorption spectra accurately (even without fully optimizing all model hyperparameters), (ii) the dimensionality reduction *via* PCA does not lead to loss of the most relevant spectral information, and (iii) the model reconstructs the essential features of the absorption spectra effectively.

Additional analyses for GLY, VAL, HIS, and PHE generate mean test *R*^2^ values of 0.9933, 0.9952, 0.9924, and 0.9965, respectively, confirming that the predictive performance remains consistently high across all five amino acids considered here (see Fig. 2 in the SI). For the representative ALA case, a 5-fold cross-validation was also applied to support our findings. The respective training/testing resulted in a mean test *R*^2^ of 0.9907 and a mean test RMSE of 2402.5, while the angle-block validation along the *θ* axis gives a mean test *R*^2^ of 0.9901 and a mean test RMSE of 2527.4. These additional checks confirm that the reported performance is not restricted to a single favorable random split; see Fig. 3 and 4 in the SI. Note that the present generalization refers to previously unseen orientations and conformations within each amino-acid-specific dataset. The present work does not test extrapolation to amino-acid species outside the set considered here.

### Prediction of the absorption spectra

**Fig. 4:**
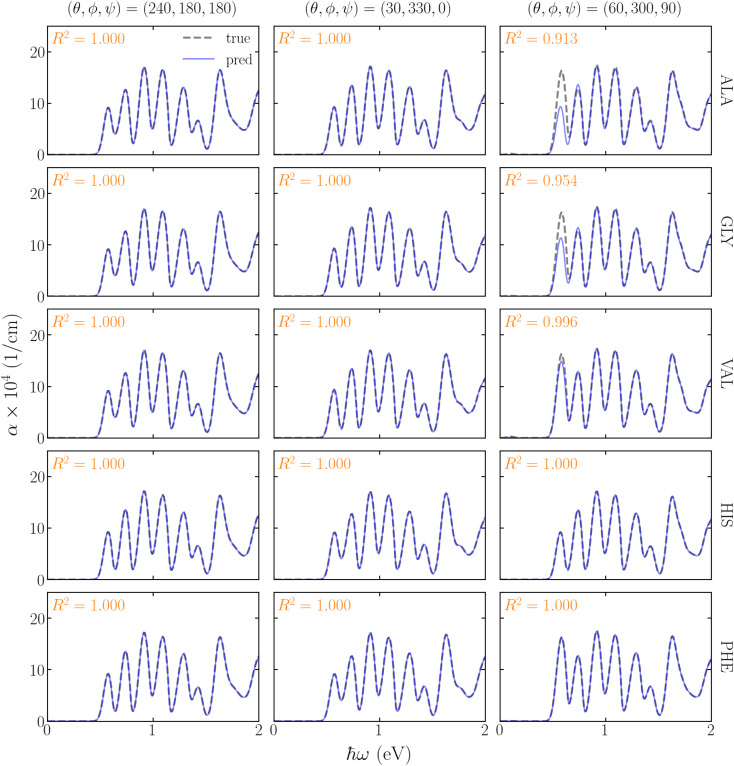
The ML-predicted (solid lines) absorption spectra obtained with the clean training-only PCA workflow for five amino acids confined in the graphene nanopore: ALA, GLY, VAL, HIS, and PHE. The dashed lines represent the DFT-calculated (ground-truth) absorption spectra. Each row corresponds to one amino acid and each column corresponds to one representative angular configuration, namely (*θ*, *ϕ*, *ψ*) = (240, 180, 180), (30, 330, 0), and (60, 300, 90). The consistently strong overlap between predicted and reference spectra demonstrates that the clean PCA workflow accurately reproduces the absorption response across both multiple molecular identities and multiple unseen orientations.

We further discuss the predictability of the trained model in predicting the absorption spectra of previously unseen (test) molecular orientations across different amino acids confined within the graphene nanopore. This is essential to evaluate, beyond the prediction capability of the model, its generalization performance when applied to configurations that were not explicitly included during training. The predicted absorption spectra are depicted in Fig. 4 together with the corresponding reference (ground-truth) spectra obtained from DFT calculations. The figure presents the clean-PCA prediction results for all five amino acids considered in this work, namely ALA, GLY, VAL, HIS, and PHE, for three representative angular configurations, namely (*θ*, *ϕ*, *ψ*) = (240, 180, 180), (30, 330, 0), and (60, 300, 90). As clearly seen, the predicted spectra for all considered amino acids and angular configurations reproduce the DFT-calculated ones across the entire photon energy range from 0 to 2 eV, demonstrating that the model has successfully learned the mapping between structural/electronic descriptors and the resulting absorption spectra. This is further quantified by the *R*^2^ scores reported in each panel, where consistently high *R*^2^ values indicate strong predictive accuracy. Accordingly, the learning model preserves the overall spectral shape, the dominant peak structure, and the relative energetic alignment of the absorption features across chemically distinct amino acids including conformational variability. This broader comparison complements the representative score-based analysis discussed above and demonstrates that the training-only PCA workflow yields consistently accurate predictions across the full set of amino acid-specific datasets.

Even in the least accurate cases, the prediction quality remains high with *R*^2^ > 0.9, and the overall spectral shape is still fairly well captured. This observation suggests that the model preserves the essential physical characteristics of the absorption process despite minor discrepancies. A closer inspection reveals that the reduction in *R*^2^ is primarily associated with deviations in the intensity of the small peak located in the low-energy region on the far left of the spectrum, as evident in the right-hand panels of the figure. Nevertheless, these deviations do not affect the predicted peak positions, which remain in excellent agreement with the DFT results. This indicates that the model accurately captures the underlying electronic transition energies, though slight inaccuracies arise in the relative amplitudes of weaker spectral features. Additional peak-based analysis for the representative ALA dataset gives a mean peak-position error of 0.0132 eV and a mean peak-intensity error of 635.4 cm^−1^ for the MLP model. This supports the interpretation that the remaining discrepancy is mainly associated with the intensity of weak excitations rather than with errors in the predicted peak locations; see Fig. 5 in the SI. Note that in sensing it is not the peak height, but rather the peak position that is used for assigning the molecule identity.

### Comparison with other baseline models

We have also compared the MLP model with simpler baseline models trained on the same descriptors and PCA-reduced targets. For the representative ALA dataset, the mean test *R*^2^ values are 0.9893 for the MLP, 0.9902 for linear regression, 0.9911 for kernel ridge regression, and 0.9932 for random forest, while the corresponding mean test RMSE values are 2611.8, 3728.4, 3551.8, and 2716.3, respectively. These results show that simpler baseline models remain competitive, although the MLP achieves the lowest RMSE among the tested models and the best peak-position and peak-intensity accuracy; see Fig. 5 and 6 in the SI. Still, the more complex MLP model is expected to be more generalizable and extendable to unseen amino acids, their sequences, and conformations.

## Conclusions

In this work, we have developed a ML workflow for the prediction of optical absorption spectra of single amino acids confined within a graphene nanopore. The approach was built upon structural, electronic, and optical data for amino acids in a 1.5 nm wide graphene nanopore from high-throughput DFT simulations. The data capture the corresponding responses of the material to the distinct molecular conformations in the pore and are molecule specific. The hybrid DFT and ML framework could establish a direct mapping between molecular configurations and the resulting absorption spectra. It demonstrated consistently high predictive performance across all amino acids and molecular configurations considered here, reproducing the absorption spectra over the entire photon energy range from 0 to 2 eV for previously unseen orientations within each amino-acid-specific dataset. Any observed discrepancies were associated with small inaccuracies in the intensity of the lowest energy peak, whereas the peak positions remain consistently and accurately predicted. Note that the spectral peak positions and not the intensities are the dominant features used for molecular identification in nanopore-based optical sensing. Furthermore, the model remains robust under both 5-fold cross-validation and stricter angle-block validation, indicating that it captures the underlying physical relationships governing the interaction between confined molecules and the nanopore without relying on a single favorable random split. The ability to reproduce all characteristic absorption peak positions suggests that the learned representation preserves essential information about electronic transitions, which is critical for distinguishing among amino acids. This hybrid strategy significantly reduces the computational cost associated with direct very demanding high-throughput DFT simulations. Although the framework was developed for graphene nanopores and amino acids it can be transferred and extended to other materials and biomolecules. By replacing repeated DFT calculations with a trained neural network, the workflow enables rapid and scalable predictions while retaining high accuracy. Accordingly, the combination of DFT and ML not only accelerates the exploration of the configuration space while retaining DFT accuracy, but also provides a practical pathway towards real-time and high-throughput optical biosensing applications using nanopores.

## Conflicts of interest

There are no conflicts to declare.

## Supplementary Material

RA-OLF-D6RA03783F-s001

## Data Availability

The entire workflow and code supporting this study are deposited in a persistent public repository at Zenodo and are available at https://doi.org/10.5281/zenodo.19893594. A representative dataset used for training and testing the machine learning model, sufficient to reproduce the key results and conclusions of this study, is also available in the same repository under the above DOI. For broader accessibility, the aforementioned workflow and code are also available at https://github.com/RWTH-CompBioTech/Nanopore-ML-Optical and https://github.com/llliphys/Nanopore-ML-Optical. Supplementary information (SI): additional figures supporting the discussion on PCA leakage, cross-validation, stricter angle-block validation, baseline-model comparisons, all-molecule performance trends, and peak-based evaluation. See DOI: https://doi.org/10.1039/d6ra03783f.

## References

[cit1] Kasianowicz J. J., Brandin E., Branton D., Deamer D. W. (1996). Characterization of individual polynucleotide molecules using a membrane channel. Proc. Natl. Acad. Sci. U. S. A..

[cit2] Dekker C. (2007). Solid-state nanopores. Nat. Nanotechnol..

[cit3] Branton D. (2008). *et al.*, The potential and challenges of nanopore sequencing. Nat. Biotechnol..

[cit4] Deamer D., Akeson M., Branton D. (2016). Three decades of nanopore sequencing. Nat. Biotechnol..

[cit5] Stoddart D., Heron A. J., Mikhailova E., Maglia G., Bayley H. (2009). Single-nucleotide discrimination in immobilized DNA oligonucleotides with a biological nanopore. Proc. Natl. Acad. Sci..

[cit6] Derrington I. M., Butler T. Z., Collins M. D., Manrao E., Pavlenok M., Niederweis M., Gundlach J. H. (2010). Nanopore DNA sequencing with MspA. Proc. Natl. Acad. Sci. U. S. A..

[cit7] Eisenstein M. (2012). Oxford Nanopore announcement sets sequencing sector abuzz. Nat. Biotechnol..

[cit8] Jain M., Olsen H. E., Paten B., Akeson M. (2016). The Oxford Nanopore MinION: delivery of nanopore sequencing to the genomics community. Genome Biol..

[cit9] Li J., Stein D., McMullan C., Branton D., Aziz M. J., Golovchenko J. A. (2001). Ion-beam sculpting at nanometre length scales. Nature.

[cit10] Storm A. J., Chen J. H., Ling X. S., Zandbergen H. W., Dekker C. (2003). Fabrication of solid-state nanopores with single-nanometre precision. Nat. Mater..

[cit11] Schneider G. F., Kowalczyk S. W., Calado V. E. (2010). others DNA translocation through graphene nanopores. Nano Lett..

[cit12] Feng J., Liu K., Bulushev R. D. (2015). others Identification of single nucleotides in MoS2 nanopores. Nat. Nanotechnol..

[cit13] Yu R.-J., Lu S.-M., Xu S.-W., Li Y.-J., Xu Q., Ying Y.-L., Long Y.-T. (2019). Single molecule sensing of amyloid-β aggregation by confined glass nanopores. Chem. Sci..

[cit14] Liu S.-C., Ying Y.-L., Li W.-H. O., Wan Y.-J., Long Y.-T. (2021). Snapshotting the transient conformations and tracing the multiple pathways of single peptide folding using a solid-state nanopore. Chem. Sci..

[cit15] Wang S., Ying Y.-L., Long Y.-T. (2025). Exploring a solid-state nanopore approach for single-molecule protein detection from single cells. Chem. Sci..

[cit16] Chen K.-L., Ying Y.-L., Ewing A. G., Long Y.-T. (2025). Nanopipette Electrochemistry. Chem. Rev..

[cit17] Wanunu M. (2012). Nanopores: A journey towards DNA sequencing. Phys. Life Rev..

[cit18] Shekar S., Niedzwiecki D. J., Chien C.-C., Ong P., Fleischer D. A., Lin J., Rosenstein J. K., Drndić M., Shepard K. L. (2016). Measurement of DNA Translocation Dynamics in a Solid-State Nanopore at 100 ns Temporal Resolution. Nano Lett..

[cit19] Rang F. J., Kloosterman W. P., de Ridder J. (2018). From squiggle to basepair: computational approaches for improving nanopore sequencing read accuracy. Genome Biol..

[cit20] Taniguchi M. (2020). Combination of Single-Molecule Electrical Measurements and Machine Learning for the Identification of Single Biomolecules. ACS Omega.

[cit21] Mittal S., Jena M. K., Pathak B. (2024). Machine learning empowered next generation DNA sequencing: perspective and prospectus. Chem. Sci..

[cit22] Chang S., Huang S., Liu H., Zhang P., Liang F., Akahori R., Li S., Gyarfas B., Shumway J., Ashcroft B., He J., Lindsay S. (2012). Chemical recognition and binding kinetics in a functionalized tunnel junction. Nanotechnology.

[cit23] Krstić P., Ashcroft B., Lindsay S. (2015). Physical model for recognition tunneling. Nanotechnology.

[cit24] Diaz Carral A., Shekar Sarap C., Liu K., Radenovic A., Fyta M. (2019). 2D MoS2 nanopores: ionic current blockade height for clustering DNA events. 2D Mater..

[cit25] Diaz Carral A., Ostertag M., Fyta M. (2021). Deep learning for nanopore ionic current blockades. J. Phys. Chem..

[cit26] Mittal S., KumarÂ Jena M., Pathak B. (2025). A hybrid supervised and unsupervised machine learning approach for identifying nucleoside drugs using nanopore readouts. Nanoscale.

[cit27] Jena M. K., Roy D., Pathak B. (2022). Machine Learning Aided Interpretable Approach for Single Nucleotide-Based DNA Sequencing using a Model Nanopore. J. Phys. Chem. Lett..

[cit28] Mittal S., Manna S., Pathak B. (2022). Machine Learning Prediction of the Transmission Function for Protein Sequencing with Graphene Nanoslit. ACS Appl. Mater. Interfaces.

[cit29] Mittal S., Jena M. K., Pathak B. (2023). Protein Sequencing with Artificial Intelligence: Machine Learning Integrated Phosphorene Nanoslit. Chem.–Eur. J..

[cit30] Jena M. K., Pathak B. (2023). Development of an Artificially Intelligent Nanopore for High-Throughput DNA Sequencing with a Machine-Learning-Aided Quantum-Tunneling Approach. Nano Lett..

[cit31] Jena M. K., Mittal S., Manna S. S., Pathak B. (2023). Deciphering DNA nucleotide sequences and their rotation dynamics with interpretable machine learning integrated C3N nanopores. Nanoscale.

[cit32] Pandit S., Jena M. K., Mittal S., Pathak B. (2024). Machine Learning Prediction and Classification of Transmission Functions for Rapid DNA Sequencing in a Hybrid Nanopore. ACS Appl. Nano Mater..

[cit33] Mittal S., Manna S., Jena M. K., Pathak B. (2023). Artificial intelligence aided recognition and classification of DNA nucleotides using MoS_2_ nanochannels. Digital Discovery.

[cit34] Tabatabaei S. K., Pham B., Pan C., Liu J., Chandak S., Shorkey S. A., Hernandez A. G., Aksimentiev A., Chen M., Schroeder C. M., Milenkovic O. (2022). Expanding the Molecular Alphabet of DNA-Based Data Storage Systems with Neural Network Nanopore Readout Processing. Nano Lett..

[cit35] Li L., Fyta M. (2026). Dielectric response of graphene and MoS2 nanopores in the detection of single amino acids. npj 2D Mater. Appl..

[cit36] Pedregosa F. (2011). *et al.*, Scikit-learn: Machine Learning in Python. J. Mach. Learn. Res..

[cit37] Paszke A. et al. , PyTorch: an Imperative Style, High-Performance Deep Learning Library, 2019, pp. 8024–8035

