# Supplementary material for: Learning biomolecular absorption spectra in graphene nanopores
Source: RSC Adv. 2026 Jul 29;16(39):43735–43. doi: 10.1039/d6ra03783f (PMC13425460; doi:10.1039/d6ra03783f)
Supplement: Supplementary file 1 [file RA-016-D6RA03783F-s001.pdf]

Supplementary Information (SI) for RSC Advances.
This journal is © The Royal Society of Chemistry 2026

Supplemental Material for "Learning biomolecular absorption spectra in graphene nanopores"

Longlong Li*,†,‡ and Maria Fyta†,‡

†*Computational Biotechnology, RWTH Aachen University, Worrignerweg 3, 52074, Aachen, Germany*

‡*Center for Computational Life Sciences (CCLS), RWTH Aachen University, Pauwelsstrasse 19, 52072, Aachen, Germany*

E-mail: l.li@biotec.rwth-aachen.de

This Supplemental Material presents the additional validation and comparison figures cited in the revised manuscript and in the response letter. These figures support the added discussion on PCA leakage, cross-validation, stricter angle-block validation, baseline-model comparisons, all-molecule performance trends, and peak-based evaluation.

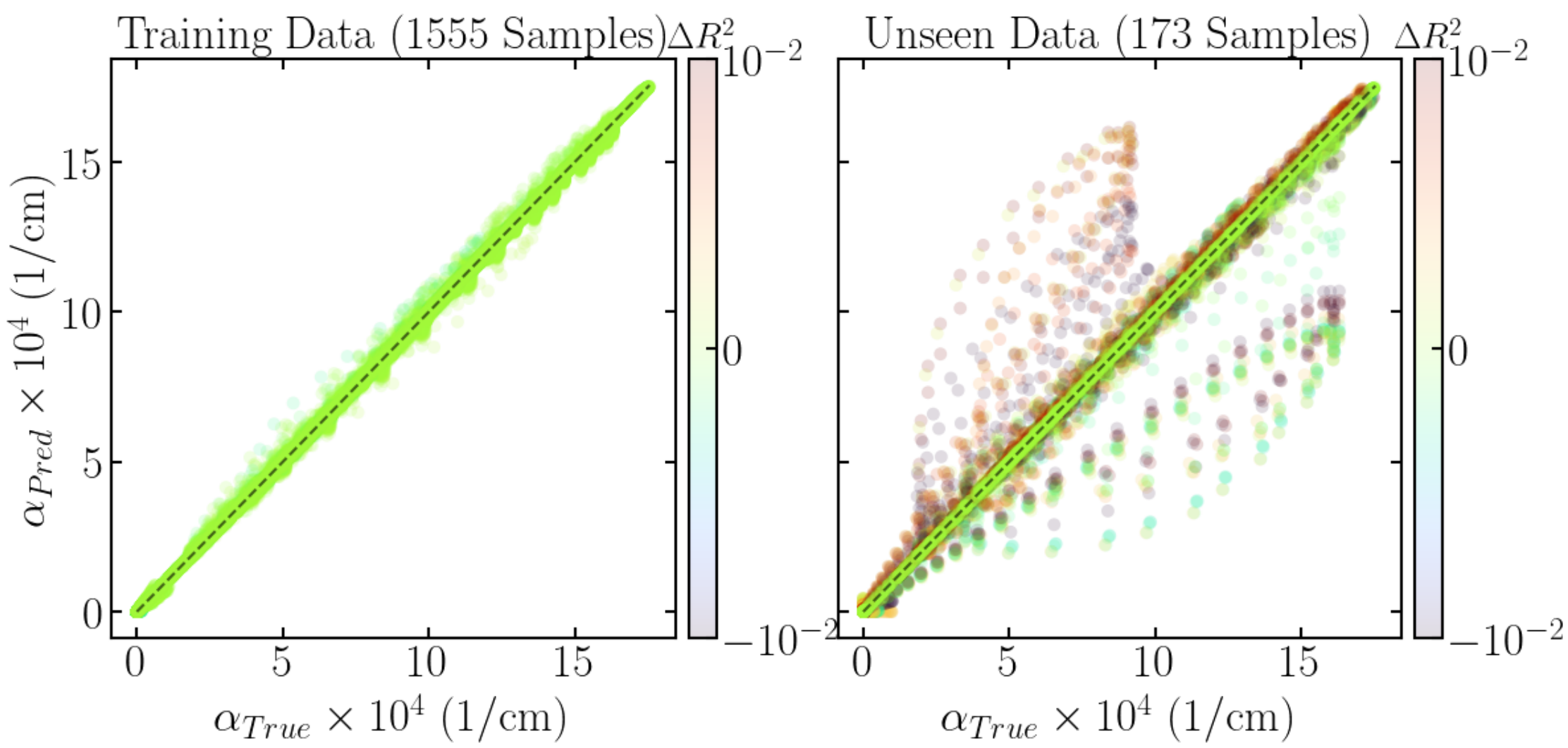

Figure 1: Sample-resolved visualization of the difference between the PCA-clean and PCA-leaky workflows for the representative ALA dataset. The two panels show the training and test data, respectively, in the same absorption-coefficient coordinates used for the main model-score plots, while the colormap encodes $\Delta R^2 = R^2_{\text{leaky}} - R^2_{\text{clean}}$. The very small color scale around zero illustrates that the two preprocessing workflows lead to nearly indistinguishable predictive performance, supporting the statement that PCA leakage does not materially affect the reported results. Note that leaky refers to a workflow in which preprocessing and the PCA is applied to the full dataset.

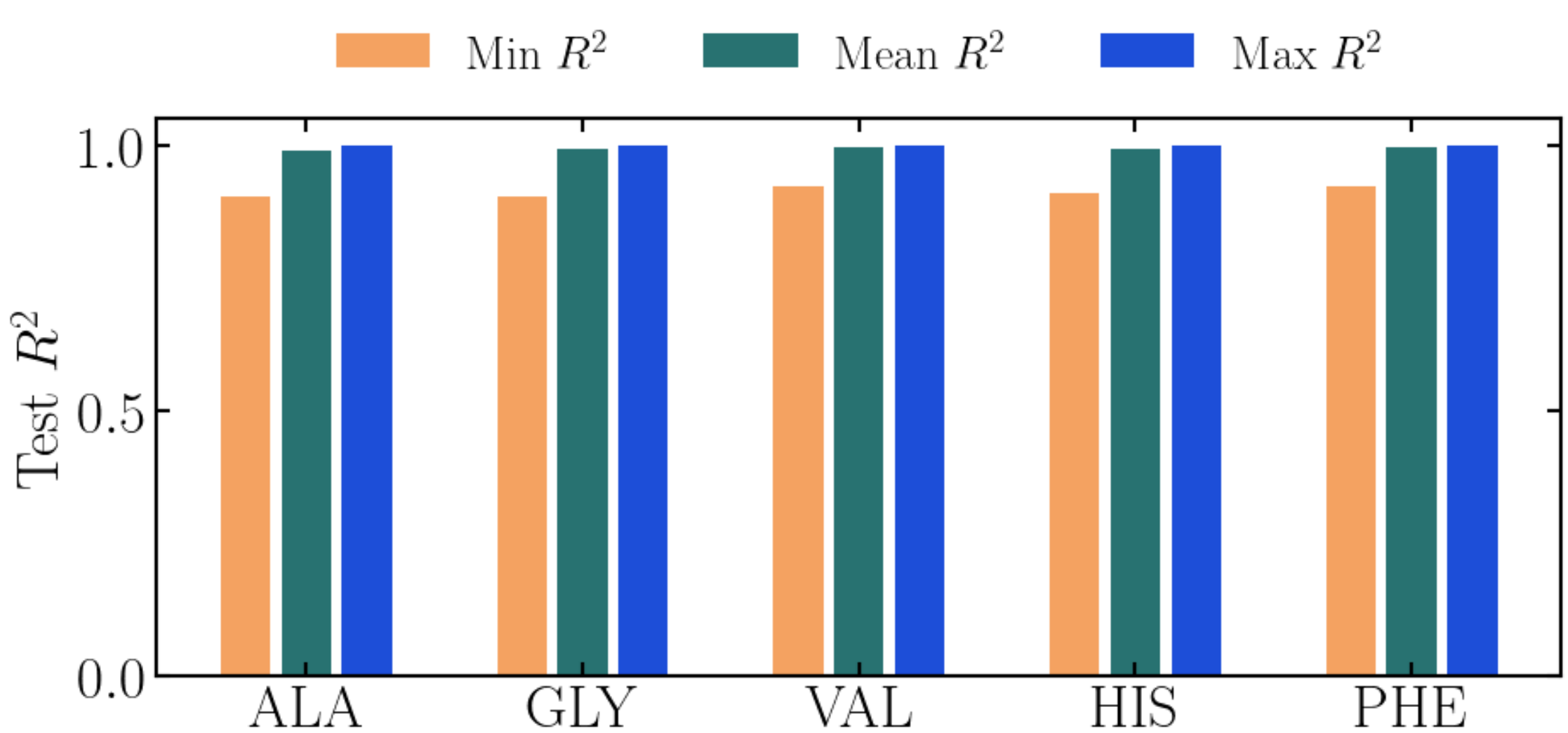

Figure 2: Per-molecule summary of the test-set R^2 values across the five amino acids considered in the study. For each amino acid, the minimum, mean, and maximum sample-level test R^2 values are shown. This figure supports the revised discussion that the predictive performance remains consistently high across ALA, GLY, VAL, HIS, and PHE.

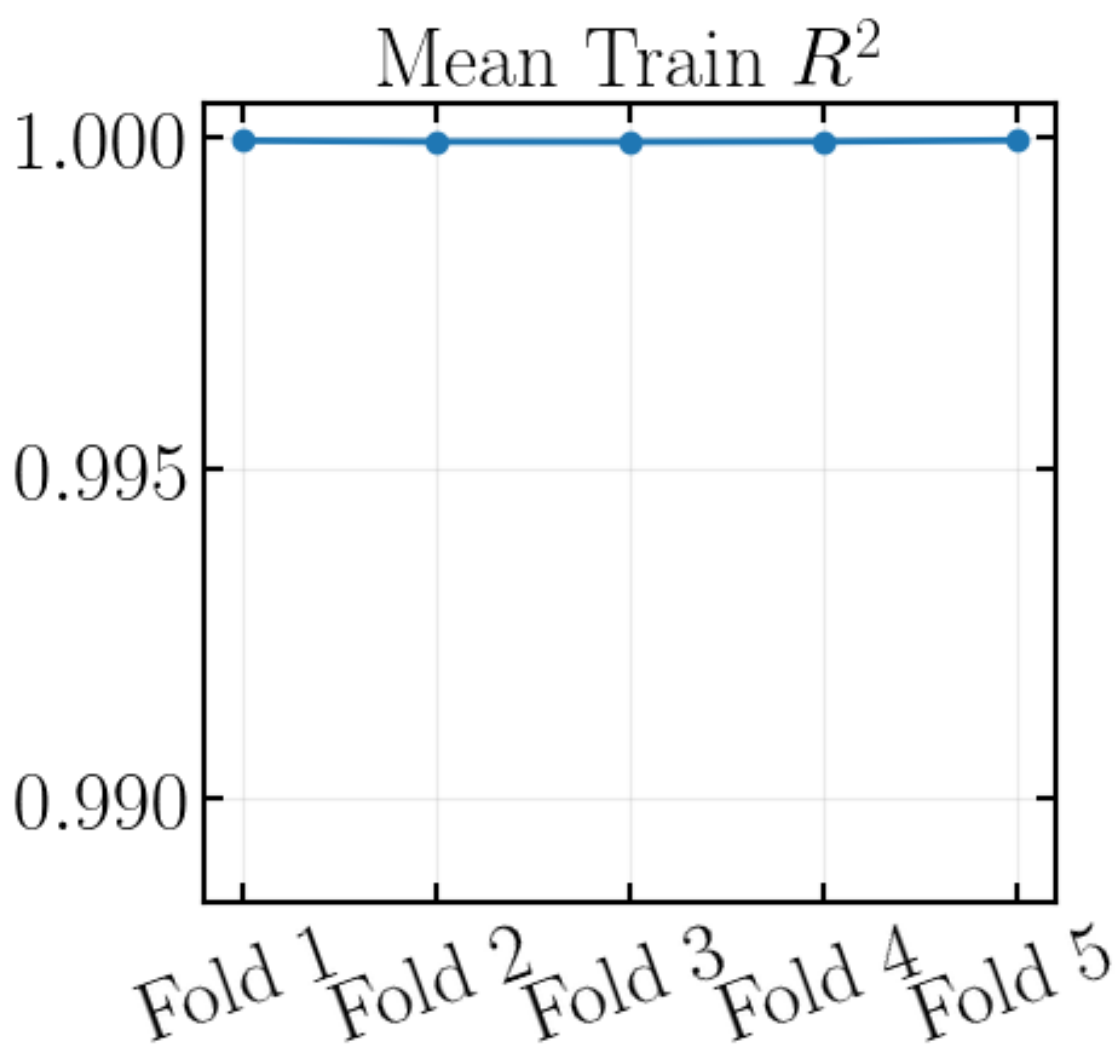

Figure 3: Five-fold cross-validation results for the representative ALA dataset. The left panel reports the training R^2 values and the right panel reports the test R^2 values for each fold. The consistently high test scores across folds support the revised claim that the model performance is not tied to one favorable random split.

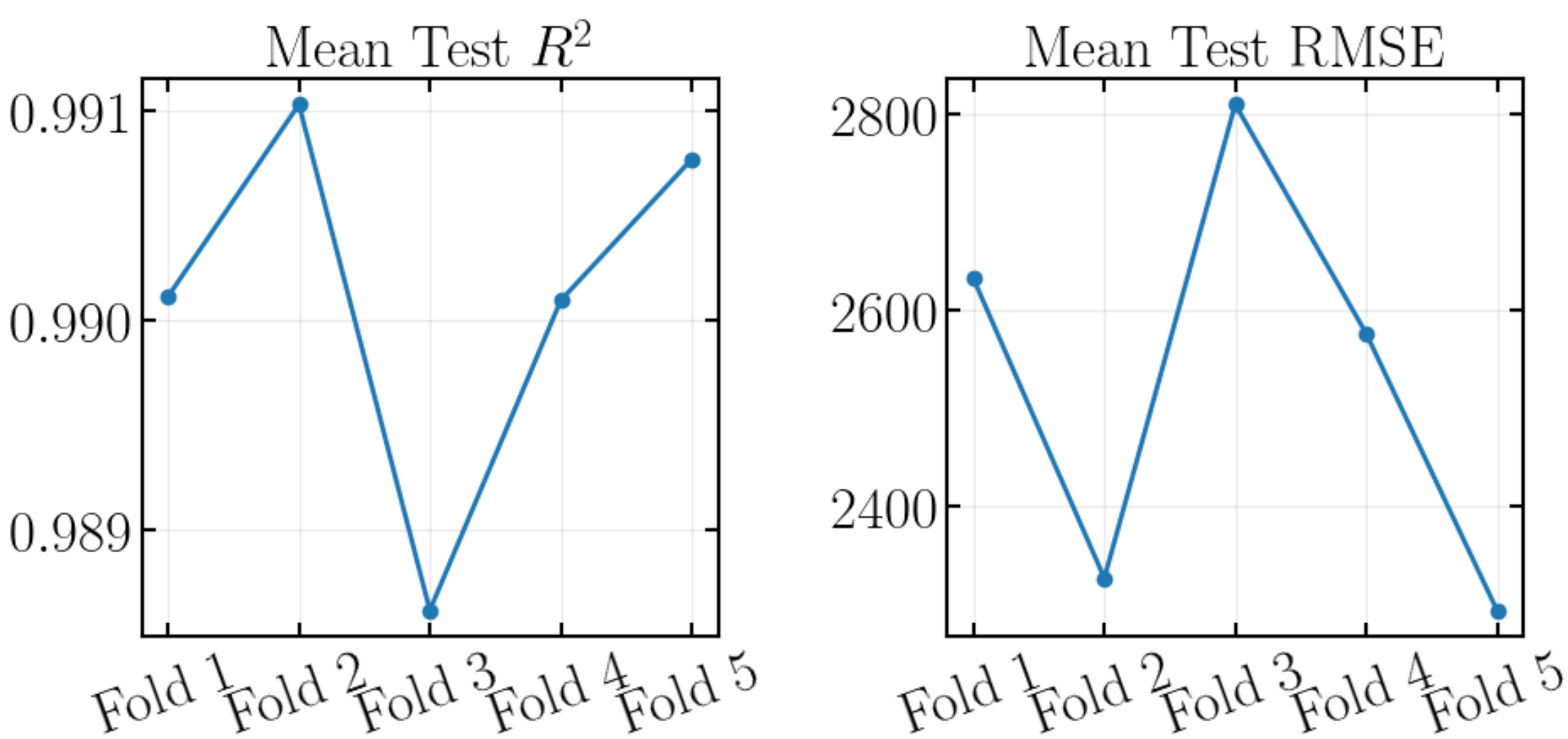

Figure 4: Angle-block validation results for the representative ALA dataset, obtained with `GroupKFold` by withholding entire groups of one angular coordinate from the training set. The figure reports fold-wise test R^2 and test RMSE values and demonstrates that the model remains accurate even under this stricter split.

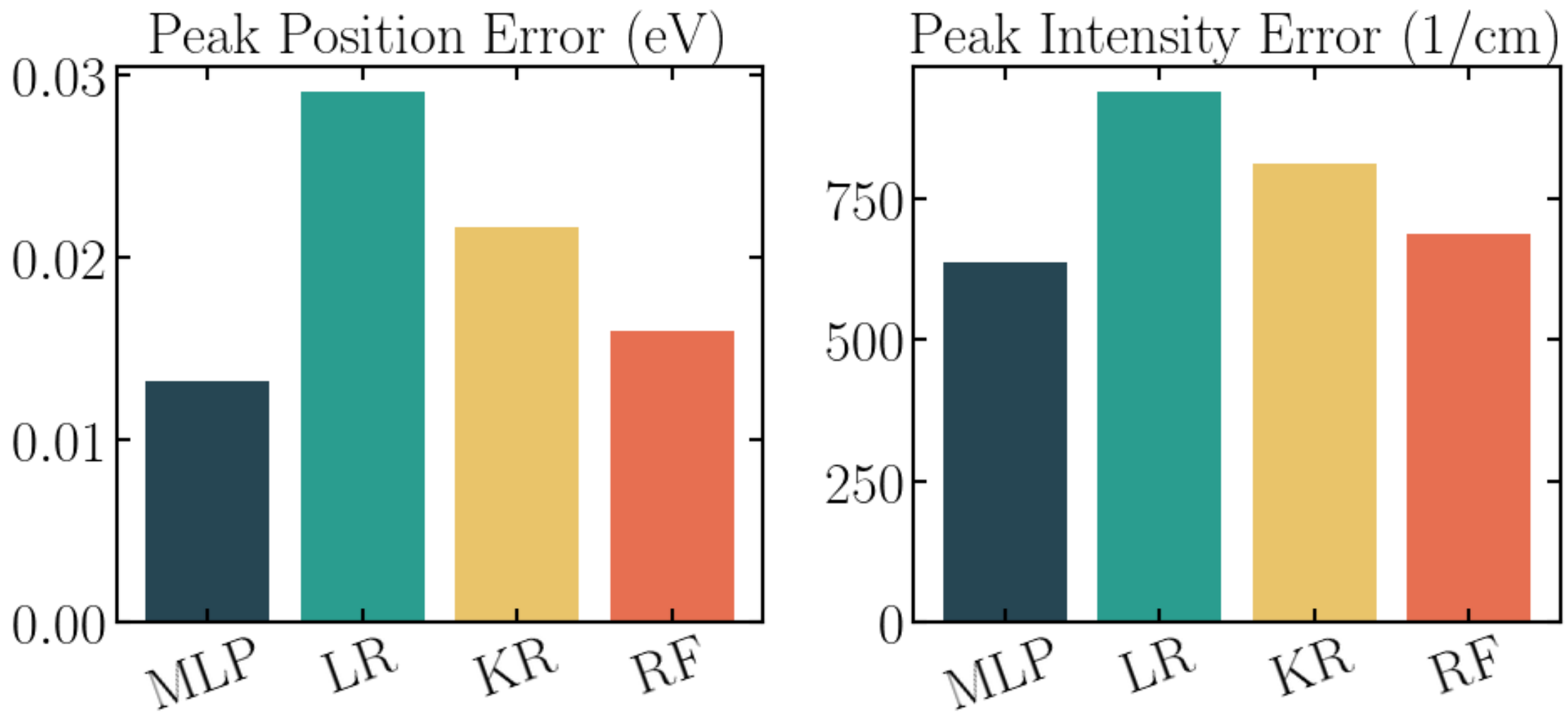

Figure 5: Peak-focused comparison of the representative ALA dataset across the MLP and the simpler baseline models (LR: linear regression; KR: kernel-ridge regression; RF: random forest) for the same data preprocessing and split. The figure reports the mean peak position error and mean peak intensity error. The results support the revised interpretation that the remaining discrepancy is dominated by weak-feature intensity prediction rather than incorrect peak placement, and that the MLP performs most favorably on these peak-specific metrics.

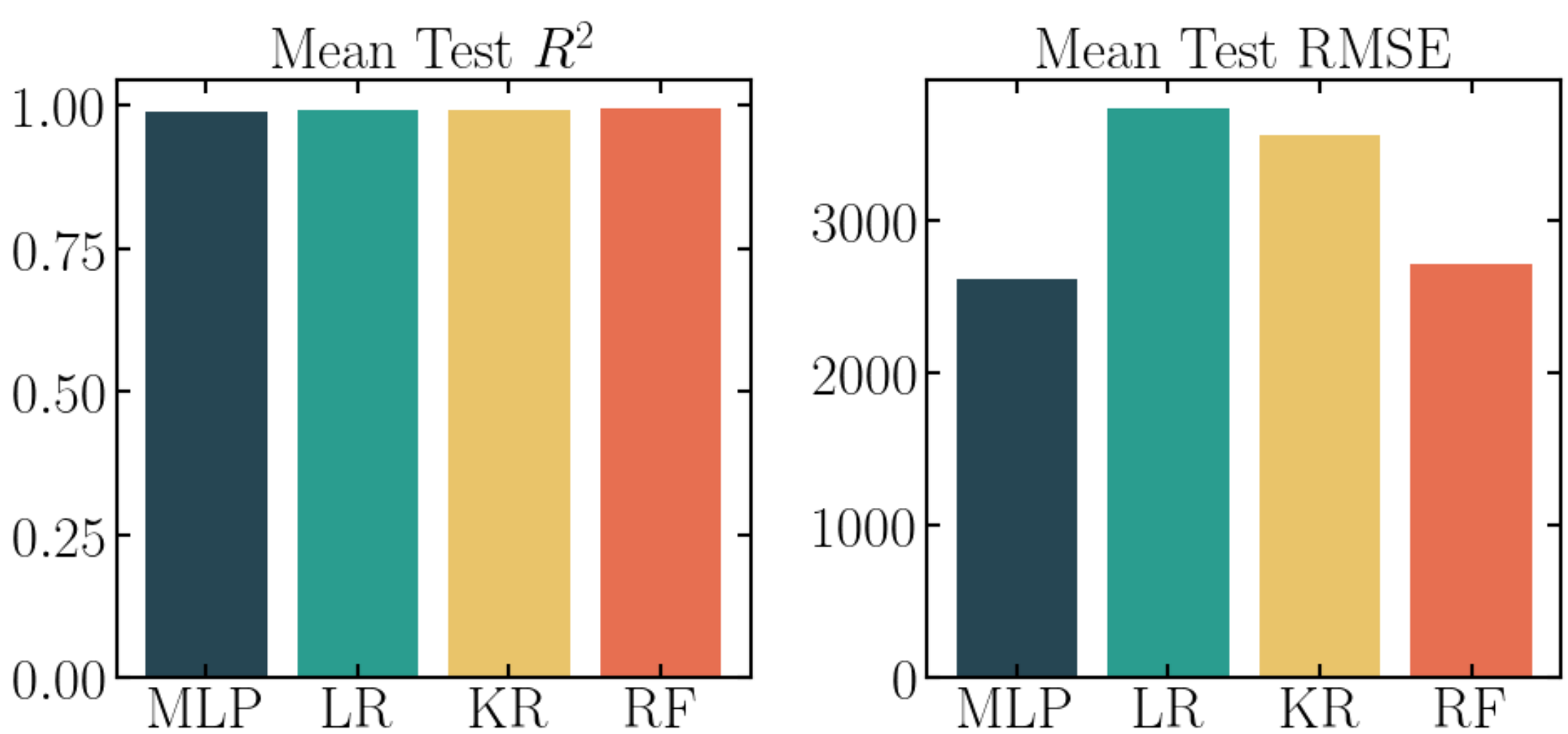

Figure 6: Comparison of the representative ALA dataset across the MLP and simpler baseline models (LR: linear regression; KR: kernel-ridge regression; RF: random forest) for the same data preprocessing and split. The figure summarizes the mean test R^2 and mean test RMSE for linear regression, kernel ridge regression, random forest, and the MLP. These data support the revised discussion that simpler models remain competitive, while the MLP remains among the strongest performers and achieves the most favorable overall balance when peak metrics are also considered.
